# Innate immune stimulation by monophosphoryl lipid A prevents chronic social defeat stress-induced anxiety-like behaviors in mice

**DOI:** 10.1186/s12974-021-02377-8

**Published:** 2022-01-07

**Authors:** Fu Li, Haitao Xiang, Yue Gu, Ting Ye, Xu Lu, Chao Huang

**Affiliations:** 1 Department of Pharmacy, Changzhou Geriatric Hospital Affiliated to Soochow University, Changzhou No.7 People’s Hospital, 288# Yanling East Road, Changzhou, 213000 Jiangsu China; 2 Department of Neurosurgery, Suzhou Kowloon Hospital, Shanghai Jiaotong University School of Medicine, #118 Wansheng Street, Suzhou, 215028 Jiangsu China; 3 Department of Pharmacology, School of Pharmacy, Nantong University, #19 Qixiu Road, Jiangsu 226001 Nantong, China

**Keywords:** Monophosphoryl lipid A, Innate immune response, Preventive effect, Pro-inflammatory cytokine

## Abstract

**Background:**

Innate immune pre-stimulation can prevent the development of depression-like behaviors in chronically stressed mice; however, whether the same stimulation prevents the development of anxiety-like behaviors in animals remains unclear. We addressed this issue using monophosphoryl lipid A (MPL), a derivative of lipopolysaccharide (LPS) that lacks undesirable properties of LPS but still keeps immune-enhancing activities.

**Methods:**

The experimental mice were pre-injected intraperitoneally with MPL before stress exposure. Depression was induced through chronic social defeat stress (CSDS). Behavioral tests were conducted to identify anxiety-like behaviors. Real-time polymerase chain reaction (PCR) and biochemical assays were employed to examine the gene and protein expression levels of pro-inflammatory markers.

**Results:**

A single MPL injection at the dose of 400 and 800 μg/kg 1 day before stress exposure prevented CSDS-induced anxiety-like behaviors, and a single MPL injection (400 μg/kg) five but not 10 days before stress exposure produced similar effect. The preventive effect of MPL on anxiety-like behaviors was also observed in CSDS mice who received a second MPL injection 10 days after the first MPL injection or a 4 × MPL injection 10 days before stress exposure. MPL pre-injection also prevented the production of pro-inflammatory cytokines in the hippocampus and medial prefrontal cortex in CSDS mice, and inhibiting the central immune response by minocycline pretreatment abrogated the preventive effect of MPL on CSDS-induced anxiety-like behaviors and pro-inflammatory cytokine productions in the brain.

**Conclusions:**

Pre-stimulation of the innate immune system by MPL can prevent chronic stress-induced anxiety-like behaviors and neuroinflammatory responses in the brain in mice.

## Background

Anxiety, which is defined as generalized anxiety disorder in human individuals, is a common psychological disorder in the modern society [1]. It can induce a variety of social burdens which may increase the morbidity of the other psychological disorders, such as depression and post-traumatic stress disorders [2, 3]. Currently, we know little of the pathogenesis of anxiety, which largely lags the development of novel strategies for the treatment of anxiety.

The microglia-mediated neuroinflammatory response in the brain, especially in the hippocampus and medial prefrontal cortex, has been demonstrated to be an important pathogenesis for psychological disorders including anxiety [4, 5]. For instance, the toll-like receptor 2/4 (TLR2/4) has been reported to mediate stress-induced microglial activation in the prefrontal cortex, which subsequently induces the development of anxiety-like behaviors in animals [6]. The inhibition of histone H3K27me3 demethylase Jumonji domain-containing protein D3 (JMJD3) or the supplementation of hydrogen sulfide can prevent lipopolysaccharide (LPS)-induced anxiety-like behaviors in animals by reducing the production of pro-inflammatory cytokines in the brain [7, 8]. Moreover, increased circulating pro-inflammatory cytokines have been observed repeatedly in patients suffering from anxiety [9–11]. Thus, the suppression of the over-production of pro-inflammatory cytokines in the brain could be potential strategy for the prevention of anxiety-associated behaviors.

In traditional opinions, microglial over-activation is indicated as a risk factor for the development of central nervous system disorders [12–14]. However, central innate immune stimulation induced by moderate activation of microglia is also neuroprotective. For example, inflammatory preconditioning with a low dose of LPS can suppress neuronal death by preventing the neuroinflammatory responses in an animal model of subarachnoid hemorrhage [15]. The low dose of LPS or macrophage-colony stimulating factor pre-treatment can also prevent epileptic seizures induced by electroconvulsive shock [16], cognitive impairment following surgery [17], neuronal damage induced by cerebral ischemia [18, 19], and the development of depression-like behaviors in chronically stressed mice [20, 21] possibly by suppressing the over-production of pro-inflammatory cytokines. These results demonstrate that central innate immune stimulation may be a potential strategy for the prevention of central nervous system disorders. However, to date, whether this strategy is capable of preventing the development of anxiety-like behaviors in animals remains unclear. In the present study, we designed a series of experiments to address this issue.

In past studies, the role of innate immune stimulation in neuronal protection is investigated using low dose of LPS pretreatment [16–19]. However, LPS, especially when administered at a relatively high dosage, may produce detrimental actions in the body, including the induction of fever and sickness behavior [22, 23]. We thus used another immune stimulant that derived from Salmonella minnesota R595 [24] and possesses unique immunomodulatory properties, including lacks of the undesirable actions of LPS, monophosphoryl lipid A (MPL) [25, 26], to examine the effect of innate immune pre-stimulation on chronic stress-induced anxiety-like behaviors in mice. We found that pre-mobilization of the innate immune response by either single or repeated MPL injections prevented chronic social defeat stress (CSDS)-induced anxiety-like behaviors and neuroinflammatory responses in the brain in mice, both of which were abrogated by innate immune inhibition. These findings may pay the way to develop novel strategies for the prevention of anxiety.

## Materials and methods

### Animals

Six-week-old male C57BL6/J mice and 8-week-old male and female CD1 mice were purchased from Beijing Vital River Laboratory Animal Technology Co., Ltd. (Beijing, China). The female CD1 mice were used to induce aggressive behaviors in male CD1 mice. Male CD1 mice would attack the intruder mice when a male C57BL6/J intruder came in and a female CD1 sexual partner was removed [27]. Mice were housed five per cage under standard vivarium conditions (12-h light/dark cycle, lights on from 07:00 to 19:00, 23 ± 1 °C ambient temperature, and 55 ± 10% relative humidity) for 1 week with free access to food and water. Experiments involving animals were approved by the University Animal Ethics Committee of Nantong University (Permit Number: 2110836) and were conducted in accordance with internationally accepted guidelines for the use of animals in toxicology as adopted by the Society of Toxicology in 1999.

### Drugs

MPL is the product of Sigma (Saint Louis, MO, USA). Minocycline was purchased from Selleck (Shanghai, China). The MPL was dissolved in dimethyl sulfoxide (DMSO) as a stock solution, and was diluted to a final concentration 100 μg/mL using the Ringer’s solution. The minocycline was dissolved in di-H_2_O as a stock solution.

### Pharmacological treatment and behavioral procedures

**Fig. 1:**
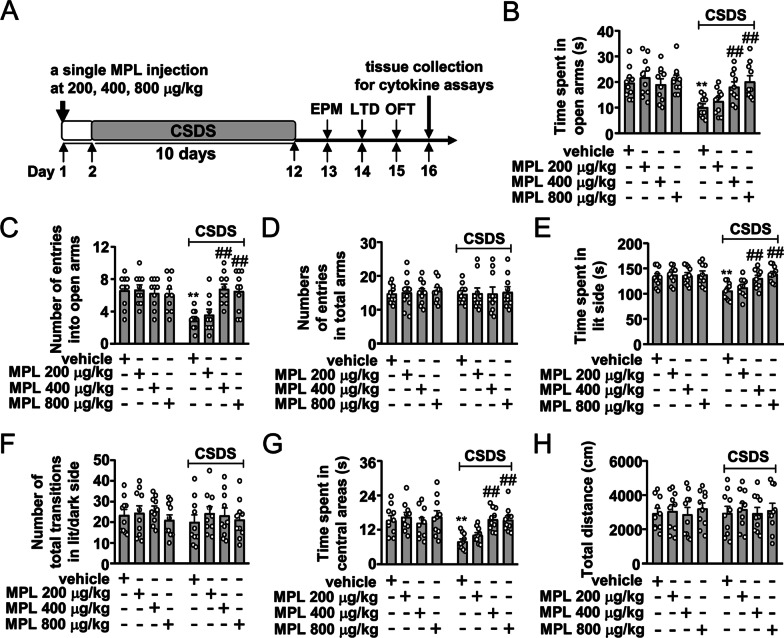
Effect of different dosages of MPL pre-injection on CSDS-induced anxiety-like behaviors in mice. **A** Timeline for the evaluation of the preventive effect of a single MPL pre-injection on CSDS-induced anxiety-like behaviors in mice. **B‒D** Quantitative analysis showing the effect of a single MPL injection (200, 400, and 800 μg/kg) 1 day before stress exposure on the time spent in open arms (**B**), the number of entries into open arms (**C**), and the number of entries into total arms (**D**) in the EPM test in mice treated with or without CSDS and/or MPL (*n* = 10, **p < 0.01 vs. vehicle; ##*p* < 0.01 vs. vehicle + CSDS). **E**, **F** Quantitative analysis showing the effect of the single MPL injection (200, 400, and 800 μg/kg) 1 day before stress exposure on the time spent in lit side (E) and the number of total transitions in both lit and dark side (F) in the LDT in mice treated with or without CSDS and/or MPL (*n* = 10, **p < 0.01 vs. vehicle; ##*p* < 0.01 vs. vehicle + CSDS). **G, H** Quantitative analysis showing the effect of the single MPL injection (200, 400, and 800 μg/kg) 1 day before stress exposure on the time spent in the center region of the open field (G) and the total distance (H) in the OFT in mice treated with or without CSDS and/or MPL (*n* = 10, **p < 0.01 vs. vehicle; ##*p* < 0.01 vs. vehicle + CSDS). Data are shown as mean ± SEM. Comparisons were made by two-way ANOVA with (**B**, **C**, **E**, **G**) or without (**D**, **F**, **H**) Bonferroni test

**Fig. 2:**
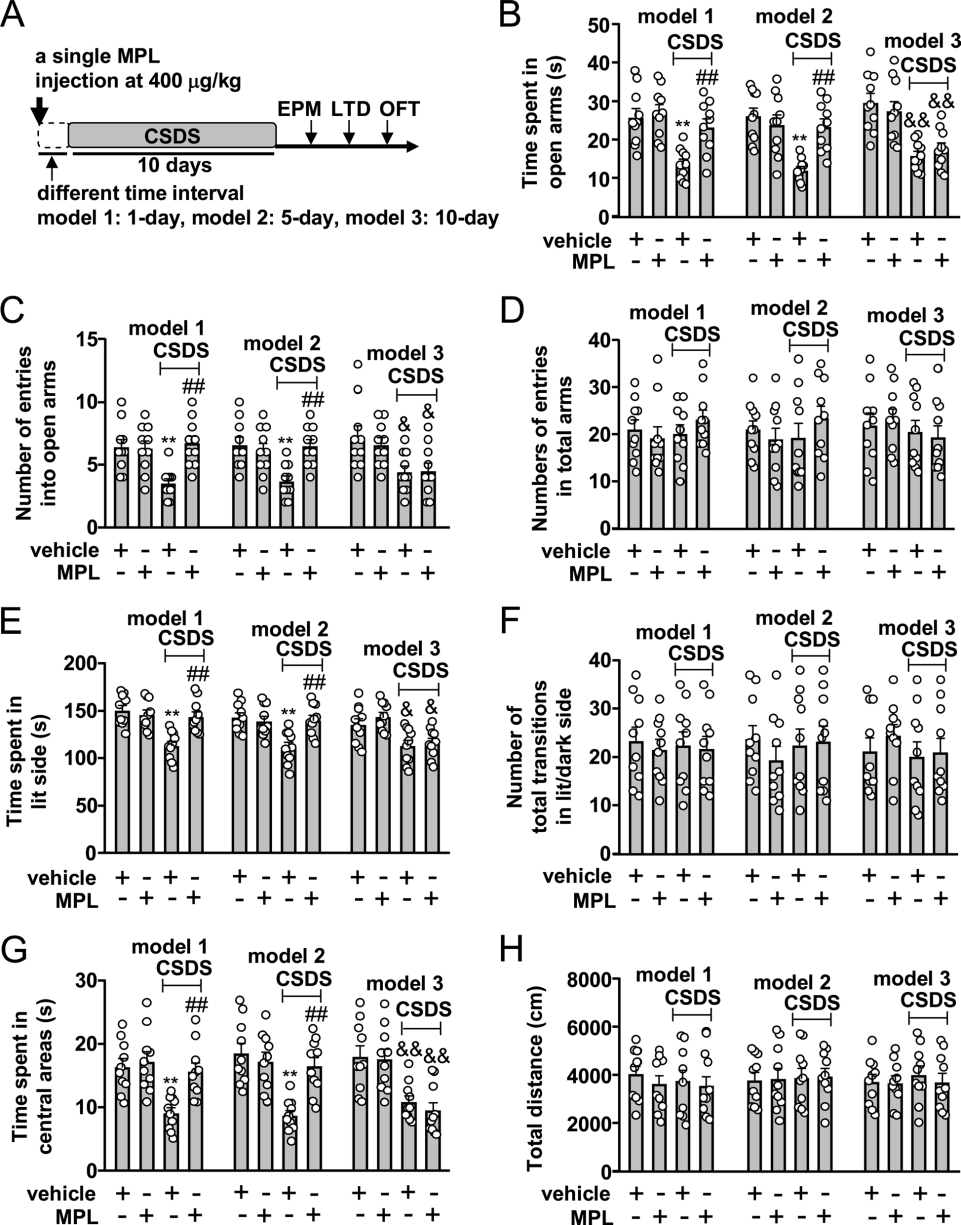
Influence of time interval on the preventive effect of MPL on CSDS-induced anxiety-like behaviors in mice. **A** Timeline for the evaluation of the influence of time interval on the preventive effect of a single MPL pre-injection on CSDS-induced anxiety-like behaviors in mice. **B–D** Quantitative analysis showing the influence of 1, 5, or 10 days of interval between the single MPL injection (400 μg/kg) and stress exposure on the time spent in open arms (**B**), the number of entries into open arms (**C**), and the number of entries into total arms (**D**) in the EPM test in mice treated with or without CSDS and/or MPL (*n* = 10, *p < 0.05 or **p < 0.01 vs. vehicle; ##*p* < 0.01 vs. vehicle + CSDS). **E, F** Quantitative analysis showing the influence of 1, 5, or 10 days of interval between the single MPL injection (400 μg/kg) and stress exposure on the time spent in lit side (**E**) and the number of total transitions (**F**) in both lit and dark side in the LDT in mice treated with or without CSDS and/or MPL (*n* = 10, *p < 0.05 or **p < 0.01 vs. vehicle; ##*p* < 0.01 vs. vehicle + CSDS). **G, H** Quantitative analysis showing the influence of 1, 5, or 10 days of interval between the single MPL injection (400 μg/kg) and stress exposure on the time spent in the center region of the open field (**G**) and the total distance (**H**) in the OFT in mice treated with or without CSDS and/or MPL (*n* = 10, **p < 0.01 vs. vehicle; ##*p* < 0.01 vs. vehicle + CSDS). Data are shown as mean ± SEM. For the results in model 1 and 2 in **B**, **C**, **E**, **G**, comparisons were made by two-way ANOVA followed by Bonferroni test, and for the results in model 3 in **B**, **C**, **E**, **G**, comparisons were first made by two-way ANOVA, and then made a further comparison between MPL pretreatment and stress exposure using *t* test according to the interaction report in ANOVA. For **D**, **F**, **H**, comparisons were only made by two-way ANOVA

**Fig. 3:**
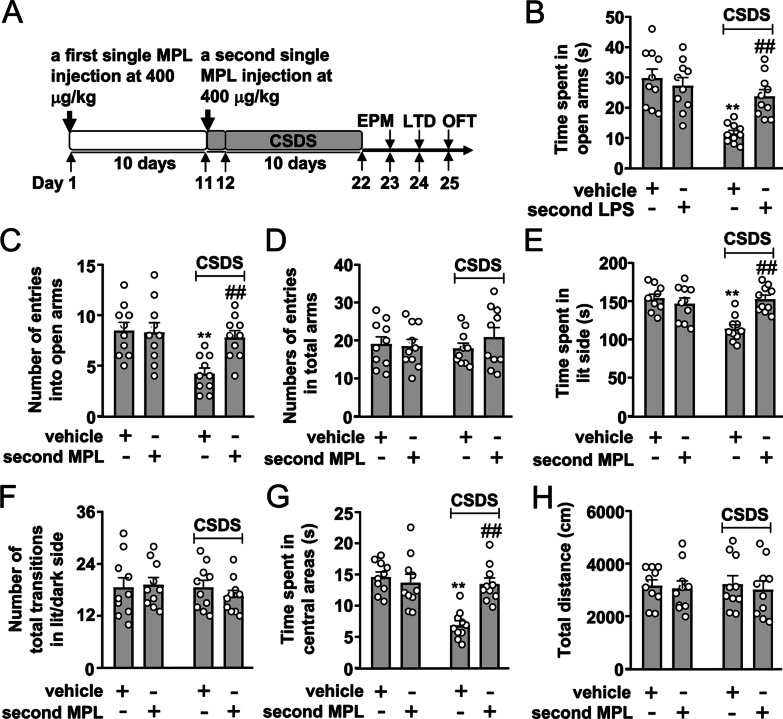
Effect of a second MPL injection 10 days after the first MPL injection on CSDS-induced anxiety-like behaviors in mice. **A** Timeline for the evaluation of the effect of the first and second MPL injection with a 10-day interval on CSDS-induced anxiety-like behaviors in mice. **B–D** Quantitative analysis showing the influence of a second MPL injection (400 μg/kg, 1 day before stress exposure) 10 days after the first MPL injection on the time spent in open arms (**B**), the number of entries into open arms (**C**), and the number of entries into total arms (**D**) in the EPM test in mice treated with or without CSDS and/or MPL (*n* = 10, **p < 0.01 vs. vehicle, in model 1 and 2; ^##^*p* < 0.01 vs. vehicle + CSDS, in model 1 and 2; ^&^*p* < 0.05 or ^&&^*p* < 0.01 vs. vehicle, in model 3). **E, F** Quantitative analysis showing the influence of a second MPL injection (400 μg/kg, 1 day before stress exposure) 10 days after the first MPL injection on the time spent in lit side (E) and the number of total transitions (F) in both lit and dark side in the LDT in mice treated with or without CSDS and/or MPL (*n* = 10, **p < 0.01 vs. vehicle, in model 1 and 2; ##*p* < 0.01 vs. vehicle + CSDS in model 1 and 2; &*p* < 0.05 vs. vehicle, in model 3). **G, H** Quantitative analysis showing the influence of a second MPL injection (400 μg/kg, 1 day before stress exposure) 10 days after the first MPL injection on the time spent in the center region of the open field (**G**) and the total distance (**H**) in the OFT in mice treated with or without CSDS and/or MPL (*n* = 10, **p < 0.01 vs. vehicle, in model 1 and 2; ^##^*p* < 0.01 vs. vehicle + CSDS, in model 1 and 2; ^&&^*p* < 0.01 vs. vehicle, in model 3). Data are shown as mean ± SEM. Comparisons were made by two-way ANOVA with (**B**, **C**, **E**, **G**) or without (**D**, **F**, **H**) Bonferroni test

**Fig. 4:**
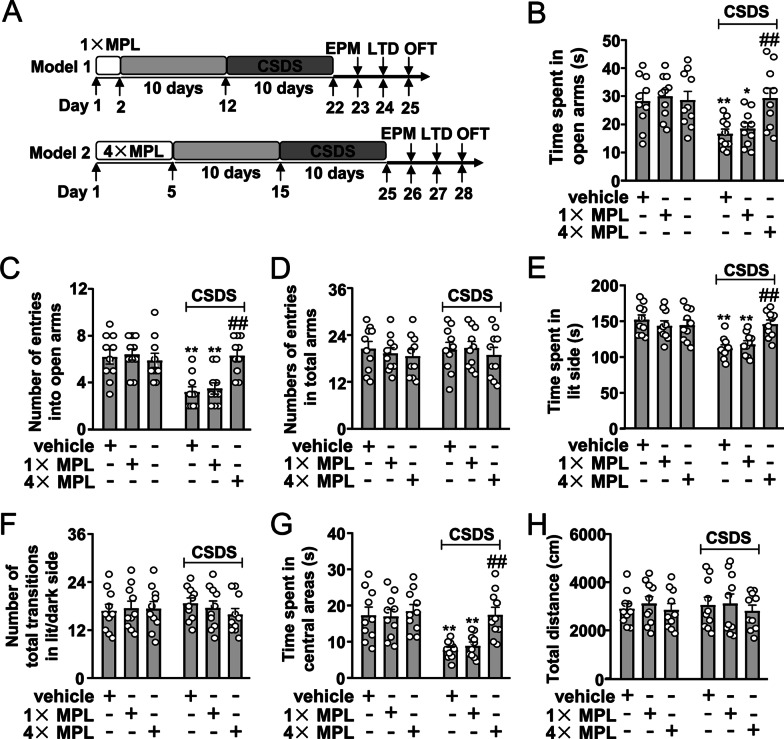
Effect of repeated MPL injection 10 days before stress exposure on CSDS-induced anxiety. **A** Timeline for the evaluation of the effect of 1 × and 4 × MPL injections on CSDS-induced anxiety-like behaviors in mice. **B–D** Quantitative analysis showing the differential effect of 1 × and 4 × MPL injections (400 μg/kg) 10 days before stress exposure on the time spent in open arms (**B**), the number of entries into open arms (**C**), and the number of entries into total arms (**D**) in the EPM test in mice treated with or without CSDS and/or MPL (*n* = 10, *p < 0.05 or **p < 0.01 vs. vehicle; ^##^*p* < 0.01 vs. vehicle + CSDS). **E**, **F** Quantitative analysis showing the differential effect of 1 × and 4 × MPL injections (400 μg/kg) 10 days before stress exposure on the time spent in lit side (**E**) and the number of total transitions (**F**) in lit and dark side in the LDT in mice treated with or without CSDS and/or MPL (*n* = 10, **p < 0.01 vs. vehicle; ##*p* < 0.01 vs. vehicle + CSDS). **G, H** Quantitative analysis showing the differential effect of 1 × and 4 × MPL injections (400 μg/kg) 10 days before stress exposure on the time spent in the center region of the open field (**G**) and the total distance (**H**) in the OFT in mice treated with or without CSDS and/or MPL (*n* = 10, **p < 0.01 vs. vehicle; ##*p* < 0.01 vs. vehicle + CSDS). Data are shown as mean ± SEM. Comparisons were made by two-way ANOVA with (**B**, **C**, **E**, **G**) or without (**D**, **F**, **H**) Bonferroni test

The dose of 200, 400, and 800 μg/kg and 1, 5, and 10 days of interval time were selected to investigate the dose- and time-dependent effect of MPL (given intraperitoneally; i.p.) on CSDS-induced anxiety-like behaviors (Fig. 1A, 2A; *n* = 10 in each group). We also evaluated the preventive effect of a second MPL injection (400 μg/kg) 10 days after the first MPL injection (Fig. 3A) or a 4 × MPL injection (400 μg/kg) 10 days before stress exposure on CSDS-induced anxiety-like behaviors (Fig. 4A; *n* = 10 in each group). To investigate the effect of MPL pre-injection on neuroinflammatory response, the mice obtained from the experiments in Fig. 1 were anesthetized with isoflurane and sacrificed by cervical dislocation immediately after the discontinuation of behavioral tests, and the fresh hippocampus and medial prefrontal cortex were separated for further detection of pro-inflammatory cytokines (*n* = 10 in each group).

Minocycline was used to investigate the role of central innate immune pre-stimulation in the preventive effect of MPL on CSDS-induced anxiety-like behaviors. Minocycline was pre-administered with a dose that was used in our past studies, 40 mg/kg (i.p.), for 2 days before MPL injection [20, 21]. Five hours after acute MPL injection the fresh brain was collected to examine the influence of minocycline on acute MPL injection-induced neuroinflammatory responses using real-time PCR (*n* = 8, in each group). For behavioral assays, minocycline was administered a continued 2 days of post-treatment after MPL injection (Fig. 6C; *n* = 10 in each group). To investigate the role of minocycline in the preventive effect of MPL on CSDS-induced neuroinflammatory responses, fresh brain tissues were separated immediately after the discontinuation of the behavioral tests (*n* = 10, in each group). Behavioral experiments were conducted during the light phase. The investigators were blinded to all of the experimental arrangement.

### CSDS

This animal model of depression was constructed according to our past studies [20, 21]. The eligibility of aggressive CD1 mouse was selected by following criteria for 3 days: the latency of CD1’s first attack was less than 90 s but longer than 5 s; the CD1 mouse attacked for at least two consecutive days during 3 day selected process. During defeat stress, each C57BL6/J mouse was exposed to a novel aggressive CD1 mouse each day for up to 10 min over a total of 10 days. After the contact, C57BL6/J mice were separated from CD1 aggressors by plastic dividers with holes during the next 24 h. To minimize physical wounds, plastic dividers were set when C57BL6/J mice displayed submissive behavior, which include immobility, trembling, crouching, fleeing, and an upright posture (usually 8–10 min was required in this study). Undefeated control mice were housed in identical cages with another C57BL/6 J mouse without being defeated with CD1 mouse and were handled throughout 10-day protocol period.

### Elevated plus maze (EPM) test

This experiment was conducted according to one of our studies [28]. The apparatus under 80 lx of illumination comprised of two opposite-facing closed arms (300 (D) × 50 (W) × 150 (H) mm), two opposite-facing open arms (300 (W) × 50 (D) mm), and a central area (50 (D) × 50 (W) mm), which were raised 50 cm above ground by a base. During test, each mouse was put in the center region facing towards the open arm. The time spent by each mouse in open arms and the number of entries into open arms and total arms were recorded for 5 min with a video camera (Anhui Zhenghua Biological instrument equipment Co. Ltd, Huaibei, China) and scored as exploratory behaviors. The arms were cleaned carefully after each trial.

### Open field test (OFT)

This experiment was conducted according to one of our studies [28]. The mice were habituated to the testing room for 20 min before the session started in a dimly environment illuminated with a red bulb (50 W) on the ceiling. During test, each mouse was placed at the center of a cubic chamber (360 (W) × 360 (H) × 360 (D) mm) and allowed to travel freely for 15 min. The time spent by each mouse in the center region of the open field and the total distance of mice were measured with an automated analyzing system (Anhui Zhenghua Biological instrument equipment Co. Ltd, Huaibei, China). The plate was cleaned carefully after each trial.

### Light–dark test (LDT)

This experiment was conducted according to one of our studies [28] using an apparatus consisting of two glass boxes (27 × 21 × 24 cm) with an interconnecting grey plastic tunnel (7 × 10 cm). One of the boxes was painted black and was weakly lit by a red 25-W bulb (0 lx). The other box was lit by a 60-W desk lamp (400 lx) placed 30 cm above the box, providing the only laboratory illumination. The floor was lined into 9 cm squares. The mice were introduced into the black compartment and observed for 5 min. The time spent by each mouse in lit side and the number of total transitions between both lit and dark side were recorded. The plate was cleaned carefully after each trial.

### Real-time polymerase chain reaction

We first extracted the total RNA in the hippocampus and medial prefrontal cortex using an RNeasy mini-kit according to manufacturer’s instructions (Qiagen, GmbH, Hilden, Germany), and then generated the first-strand of cDNA using a reverse transcription system (Promega, Madison, WI, USA). The real-time PCR was conducted with a reaction system containing 1 × Faststart SYBR Green Master Mix (Roche Molecular Biochemicals), 2 μL of diluted cDNA, 2 mMMgCl_2_, and 0.5 μM of primers: interleukin-1β (IL-1β), 5′-TGGAAAAGCGGTTTGTCTTC-3′ (F), 5′-TACCAGTTGGGGAACTCTGC-3′ (R); IL-6: 5′-AGAGATACAAAGAAATGATGGA-3′ (F), 5′-AGCTATGGTACTCCACAAGACCA-3′ (R); glyceraldehyde-3-phosphate dehydrogenase (GAPDH): 5’-GGCCTTCCGTGTTCCTAC-3’ (F), 5’-TGTCATCATATCTGGCAGGTT-3’ (R). The PCR products were detected by monitoring the increase in intensity of fluorescence emitted by the double-stranded DNA-binding dye SYBR Green. An analysis of gene expression was performed using the -ΔΔCt method. The values were normalized to GAPDH.

### Detection of cytokines

Quantitative determination of brain cytokines was determined by commercial Enzyme Linked-Immuno-Sorbent Assay kits purchased from Proteintech (Wuhan, China). The IL-1β, IL-6, and TNF-α protein were captured by the pre-coated antibodies against IL-1β, IL-6, and TNF-α on the 96-wells. Following extensive washing, another biotinylated antibody specific for IL-1β, IL-6, or TNF-α was added to detect the captured cytokine protein. For signal development, streptavidin–HRP was added, followed by a tetramethyl-benzidine reagent. Solutions containing sulfuric acid were used to stop color development and the color density which was proportional to the quantity of bound protein was measurable at 450 nm with the correction wavelength set at 630 nm.

### Statistical analysis

**Fig. 5:**
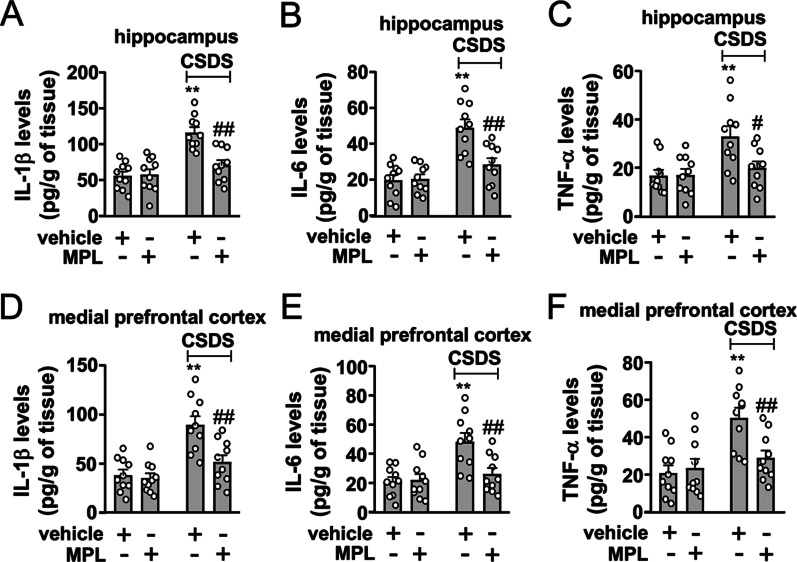
Effect of MPL pre-injection on CSDS-induced neuroinflammatory responses in the brain. **A‒F** Quantitative analysis showing the preventive effect of a single MPL injection (400 μg/kg) 1 day before stress exposure on CSDS-induced increases in the levels of IL-1β (**A** hippocampus; **D** medial prefrontal cortex), IL-6 (**B** hippocampus; **E** medial prefrontal cortex), and TNF-α (**C** hippocampus; **F** cortex) in the hippocampus and medial prefrontal cortex (*n* = 10, **p < 0.01 vs. vehicle; #*p* < 0.05 or ##*p* < 0.01 vs. vehicle + CSDS). Data are shown as mean ± SEM. Comparisons were made by two-way ANOVA followed by Bonferroni test

**Fig. 6:**
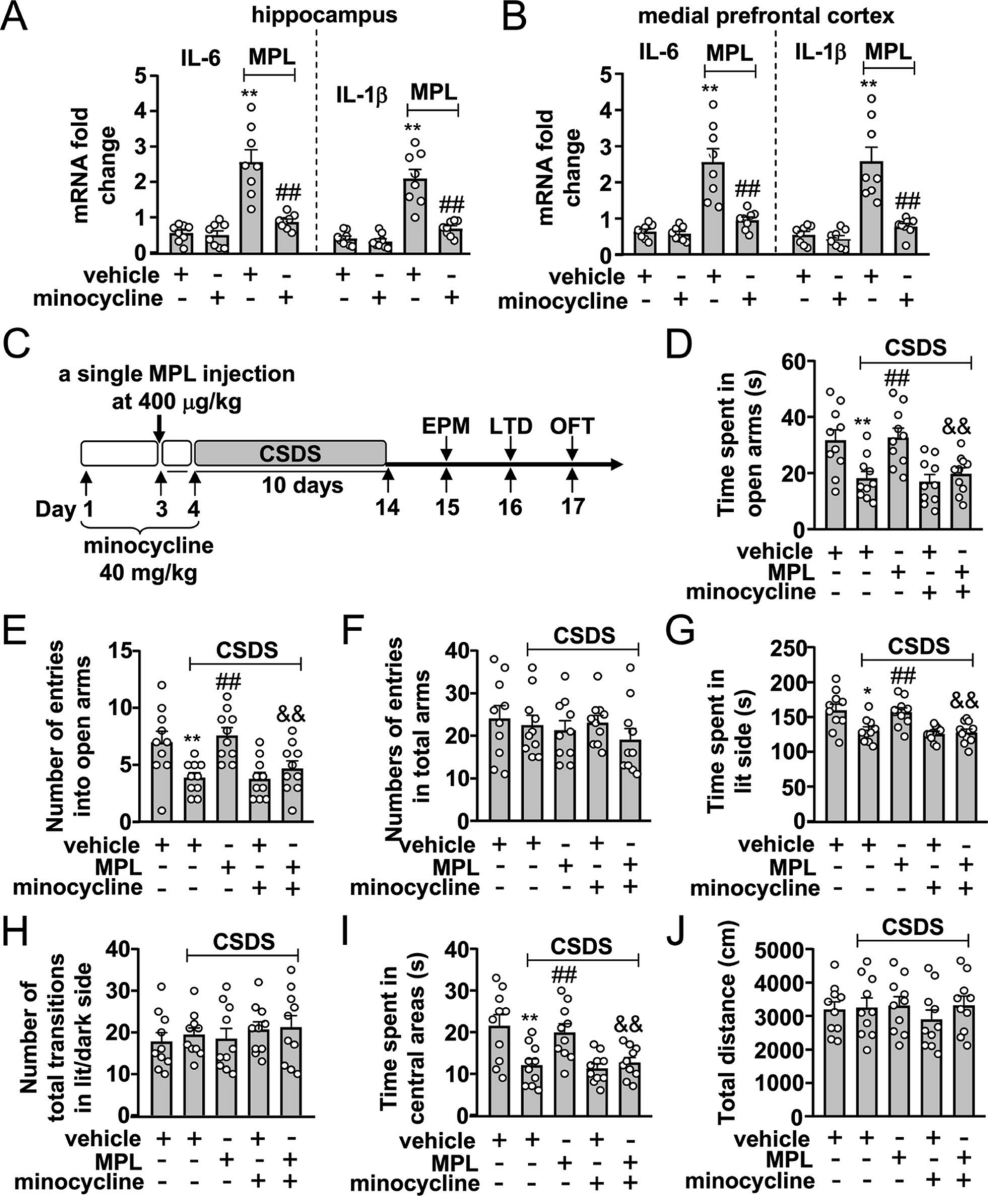
Effect of minocycline pretreatment on MPL-induced acute neuroinflammatory response and prevention of CSDS-induced anxiety-like behaviors in mice. **A**, **B** Quantitative analysis showing the preventive effect of minocycline pretreatment on acute MPL injection (400 μg/kg)-induced increases in the expression levels of IL-6 and IL-1β mRNA in the hippocampus and medial prefrontal cortex (*n* = 8, **p < 0.01 vs. vehicle; ##*p* < 0.01 vs. vehicle + MPL). **C** Timeline for the evaluation of minocycline pretreatment on the preventive effect of MPL on CSDS-induced anxiety-like behaviors in mice. **D–F** Quantitative analysis showing the effect of minocycline pretreatment on the time spent in open arms (**D**), the number of entries into open arms (**E**), and the number of entries into total arms (**F**) in the EPM test in mice treated with or without CSDS and/or MPL (*n* = 10, **p < 0.01 vs. vehicle; ##*p* < 0.01 vs. vehicle + CSDS; &&*p* < 0.01 vs. MPL + CSDS). **G, H** Quantitative analysis showing the effect of minocycline pretreatment on the time spent in lit side (**G**) and the number of total transitions (**H**) in both lit and dark side in the LDT in mice treated with or without CSDS and/or MPL (*n* = 10, *p < 0.05 vs. vehicle; ##*p* < 0.01 vs. vehicle + CSDS; &&*p* < 0.01 vs. MPL + CSDS). **I, J** Quantitative analysis showing the effect of minocycline pretreatment on the time spent in the center region of the open field (**I**) and the total distance (**J**) in the OFT in mice treated with or without CSDS and/or MPL (*n* = 10, **p < 0.01 vs. vehicle; ##*p* < 0.01 vs. vehicle + CSDS; &&*p* < 0.01 vs. MPL + CSDS). Data are shown as mean ± SEM. Comparisons were made by two-way ANOVA with (**D**, **E**, **G**, **I**) or without (**F**, **H**, **J**) Bonferroni test

Statistical analyses were performed using Graphpad Prism 8 (Graphpad Software, Inc., La Jolla, CA, USA). Differences between the mean values of the data in Figs. 1B‒H, 2B‒H (model 1 and 2), Fig. 3B‒H, 4B‒H, 5A‒F, 6A, B, 6D‒J, and 7A‒F in Table 1 were evaluated using the two-way analysis of variance (ANOVA), among which the data in Figure B, C, E, and G in Figs. 1, 2, 3, 4 and 6D, E, G, and I were made a further isolated comparison using the post-hoc Bonferroni test. When any two factors in the experiment, such as the CSDS stimulation or the vehicle/MPL treatment in model 3 in Fig. 2B‒H in Table 1, did not interact (e.g., *p* > 0.05) in the ANOVA, we used a *t* test that has been described by Wei et al. (2012) to make further comparisons between different factors [29]. *p* values < 0.05 were considered statistically significant. Data are presented as mean ± standard error of mean (SEM).

**Fig. 7:**
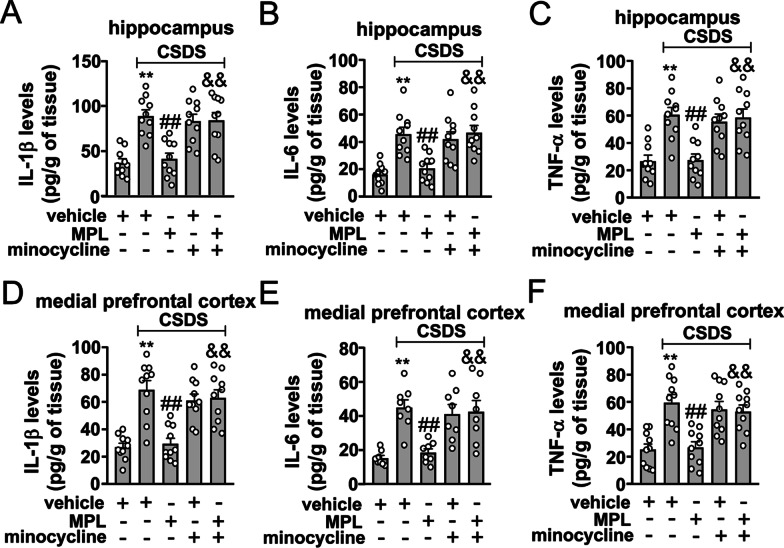
Effect of minocycline pretreatment on MPL-induced prevention of CSDS-induced neuroinflammatory responses in the brain. **A–F** Quantitative analysis showing the abrogation effect of minocycline pretreatment (40 mg/kg) on a single MPL injection (400 μg/kg, 1 day before stress exposure)-induced prevention of CSDS-induced increases in the levels of IL-1β (**A** hippocampus; **D** medial prefrontal cortex), IL-6 (**B** hippocampus; **E** medial prefrontal cortex), and TNF-α (**C** hippocampus; **F** cortex) in the hippocampus and medial prefrontal cortex (*n* = 10, **p < 0.01 vs. vehicle; ##*p* < 0.01 vs. vehicle + CSDS; &&*p* < 0.01 vs. MPL + CSDS). Data are shown as mean ± SEM. Comparisons were made by two-way ANOVA followed by Bonferroni test

**Table 1:** Statistical data for the results in each figure

Figure 1		
Figure 1B	EPM test: time spent in open arms	Significant effects: CSDS: *F*_1,72_ = 14.19, *p* < 0.001 vehicle/MPL: *F*_3,72_ = 3.10, *p* < 0.05 CSDS × vehicle/MPL interaction: *F*_3,72_ = 3.29, *p* < 0.05
Figure 1C	EPM test: number of entries into open arms	Significant effects: CSDS: *F*_1,72_ = 11.05, *p* < 0.01 vehicle/MPL: *F*_3,72_ = 3.85, *p* < 0.05 CSDS × vehicle/MPL interaction: *F*_3,72_ = 6.38, *p* < 0.001
Figure 1D	EPM test: number of entries into total arms	No significant effects: CSDS: *F*_1,72_ = 0.05, *p* = 0.82 vehicle/MPL: *F*_3,72_ = 0.09, *p* = 0.96 CSDS × vehicle/MPL interaction: *F*_3,72_ = 0.002, *p* = 0.10
Figure 1E	LDT: time spent in lit side	Significant effects: CSDS: *F*_1,72_ = 12.21, *p* < 0.001 vehicle/MPL: *F*_3,72_ = 4.30, *p* < 0.01 CSDS × vehicle/MPL interaction: *F*_3,72_ = 3.02, *p* < 0.05
Figure 1F	LDT: number of total transitions in both lit and dark side	No significant effects: CSDS: *F*_1,72_ = 0.46, *p* = 0.50 vehicle/MPL: *F*_3,72_ = 0.70, *p* = 0.56 CSDS × vehicle/MPL interaction: *F*_3,72_ = 0.17, *p* = 0.91
Figure 1G	OFT: time spent in the center region of the open field	Significant effects: CSDS: *F*_1,72_ = 11.09, *p* < 0.01 vehicle/MPL: *F*_3,72_ = 3.51, *p* < 0.05 CSDS × vehicle/MPL interaction: *F*_3,72_ = 4.13, *p* < 0.01
Figure 1H	OFT: total distance	No significant effects: CSDS: *F*_1,72_ = 0.008, *p* = 0.93, vehicle/MPL: *F*_3,72_ = 0.25, *p* = 0.86 CSDS × vehicle/MPL interaction: *F*_3,72_ = 0.03, *p* = 0.99
Figure 2		
Figure 2B: model 1	EPM test: time spent in open arms	Significant effects: CSDS: *F*_1,36_ = 16.54, *p* < 0.001 vehicle/MPL: *F*_1,36_ = 7.64, *p* < 0.01 CSDS × vehicle/MPL interaction: *F*_1,36_ = 4.48, *p* < 0.05
Figure 2C: model 1	EPM test: number of entries into open arms	Significant effects: CSDS: *F*_1,36_ = 5.06, *p* < 0.05 vehicle/MPL: *F*_1,36_ = 7.79, *p* < 0.01 CSDS × vehicle/MPL interaction: *F*_1,36_ = 8.82, *p* < 0.01
Figure 2D: model 1	EPM test: number of entries into total arms	No significant effects: CSDS: *F*_1,36_ = 0.51, *p* = 0.48 vehicle/MPL: *F*_1,36_ = 0.08, *p* = 0.78 CSDS × vehicle/MPL interaction: *F*_1,36_ = 1.42, *p* = 0.24
Figure 2E: model 1	LDT: time spent in lit side	Significant effects: CSDS: *F*_1,36_ = 15.08, *p* < 0.001 vehicle/MPL: *F*_1,36_ = 6.53, *p* < 0.05 CSDS × vehicle/MPL interaction: *F*_1,36_ = 12.13, *p* < 0.01
Figure 2F: model 1	LDT: number of total transitions in both lit and dark side	No significant effects: CSDS: *F*_1,36_ = 0.02, *p* = 0.89 vehicle/MPL: *F*_1,36_ = 0.27, *p* = 0.60 CSDS × vehicle/MPL interaction: *F*_1,36_ = 0.05, *p* = 0.83
Figure 2G: model 1	OFT: time spent in the center region of the open field	Significant effects: CSDS: *F*_1,36_ = 12.06, *p* < 0.01 vehicle/MPL: *F*_1,36_ = 8.33, *p* < 0.01 CSDS × vehicle/MPL interaction: *F*_1,36_ = 4.98, *p* < 0.05
Figure 2H: model 1	OFT: total distance	No significant effects: CSDS: *F*_1,36_ = 0.02, *p* = 0.88 vehicle/MPL: *F*_1,36_ = 0.37, *p* = 0.55 CSDS × vehicle/MPL interaction: *F*_1,36_ = 0.07, *p* = 0.79
Figure 2B: model 2	EPM test: time spent in open arms	Significant effects: CSDS: *F*_1,36_ = 13.59, *p* < 0.001 vehicle/MPL: *F*_1,36_ = 5.41, *p* < 0.05 CSDS × vehicle/MPL interaction: *F*_1,36_ = 11.92, *p* < 0.01
Figure 2C: model 2	EPM test: number of entries into open arms	Significant effects: CSDS: *F*_1,36_ = 5.41, *p* < 0.05 vehicle/MPL: *F*_1,36_ = 4.58, *p* < 0.05 CSDS × vehicle/MPL interaction: *F*_1,36_ = 9.43, *p* < 0.01
Figure 2D: model 2	EPM test: number of entries into total arms	No significant effects: CSDS: *F*_1,36_ = 0.27, *p* = 0.61 vehicle/MPL: *F*_1,36_ = 0.16, *p* = 0.69 CSDS × vehicle/MPL interaction: *F*_1,36_ = 1.54, *p* = 0.22
Figure 2E: model 2	LDT: time spent in lit side	Significant effects: CSDS: *F*_1,36_ = 8.97, *p* < 0.01 vehicle/MPL: *F*_1,36_ = 5.93, *p* < 0.05 CSDS × vehicle/MPL interaction: *F*_1,36_ = 10.22, *p* < 0.01
Figure 2F: model 2	LDT: number of total transitions in both lit and dark side	No significant effects: CSDS: *F*_1,36_ = 0.19, *p* = 0.67 vehicle/MPL: *F*_1,36_ = 0.36, *p* = 0.55 CSDS × vehicle/MPL interaction: *F*_1,36_ = 0.75, *p* = 0.39
Figure 2G: model 2	OFT: time spent in the center region of the open field	Significant effects: CSDS: *F*_1,36_ = 15.85, *p* < 0.001 vehicle/MPL: *F*_1,36_ = 6.06, *p* < 0.05 CSDS × vehicle/MPL interaction: *F*_1,36_ = 11.74, *p* < 0.01
Figure 2H: model 2	OFT: total distance	No significant effects: CSDS: *F*_1,36_ = 0.07, *p* = 0.79 vehicle/MPL: *F*_1,36_ = 0.03, *p* = 0.87 CSDS × vehicle/MPL interaction: *F*_1,36_ = 0.0001, *p* = 0.10
Figure 2B: model 3	EPM test: time spent in open arms	significant effect: CSDS: *F*_1,36_ = 34.20, *p* < 0.001 No significant effects: vehicle/MPL: *F*_1,36_ = 0.03, *p* = 0.85 CSDS × vehicle/MPL interaction: *F*_1,36_ = 0.93, *p* = 0.34
Figure 2C: model 3	EPM test: number of entries into open arms	significant effect: CSDS: *F*_1,36_ = 13.77, *p* < 0.001 No significant effects: vehicle/MPL: *F*_1,36_ = 0.14, *p* = 0.71 CSDS × vehicle/MPL interaction: *F*_1,36_ = 0.28, *p* = 0.60
Figure 2D: model 3	EPM test: number of entries into total arms	No significant effects: CSDS: *F*_1,36_ = 1.18, *p* = 0.28 vehicle/MPL: *F*_1,36_ = 0.002, *p* = 0.97 CSDS × vehicle/MPL interaction: *F*_1,36_ = 0.30, *p* = 0.59
Figure 2E: model 3	LDT: time spent in lit side	significant effect: CSDS: *F*_1,36_ = 19.96, *p* < 0.001 No significant effects: vehicle/MPL: *F*_1,36_ = 0.99, *p* = 0.33 CSDS × vehicle/MPL interaction: *F*_1,36_ = 0.23, *p* = 0.64
Figure 2F: model 3	LDT: number of total transitions in both lit and dark side	No significant effects: CSDS: *F*_1,36_ = 0.69, *p* = 0.41 vehicle/MPL: *F*_1,36_ = 0.58, *p* = 0.45 CSDS × vehicle/MPL interaction: *F*_1,36_ = 0.19, *p* = 0.67
Figure 2G: model 3	OFT: time spent in the center region of the open field	significant effect: CSDS: *F*_1,36_ = 29.00, *p* < 0.001 No significant effects: vehicle/MPL: *F*_1,36_ = 0.37, *p* = 0.55 CSDS × vehicle/MPL interaction: *F*_1,36_ = 0.13, *p* = 0.73
Figure 2H: model 3	OFT: total distance	No significant effects: CSDS: *F*_1,36_ = 0.24, *p* = 0.63 vehicle/MPL: *F*_1,36_ = 0.27, *p* = 0.60 CSDS × vehicle/MPL interaction: *F*_1,36_ = 0.16, *p* = 0.69
Figure 3		
Figure 3B	EPM test: time spent in open arms	Significant effects: CSDS: *F*_1,36_ = 21.85, *p* < 0.001 vehicle/MPL: *F*_1,36_ = 4.42, *p* < 0.05 CSDS × vehicle/MPL interaction: *F*_1,36_ = 10.07, *p* < 0.01
Figure 3C	EPM test: number of entries into open arms	Significant effects: CSDS: *F*_1,36_ = 9.79, *p* < 0.01 vehicle/MPL: *F*_1,36_ = 4.91, *p* < 0.05 CSDS × vehicle/MPL interaction: *F*_1,36_ = 6.14, *p* < 0.05
Figure 3D	EPM test: number of entries into total arms	No significant effects: CSDS: *F*_1,36_ = 0.10, *p* = 0.79 vehicle/MPL: *F*_1,36_ = 0.38, *p* = 0.54 CSDS × vehicle/MPL interaction: *F*_1,36_ = 0.86, *p* = 0.36
Figure 3E	LDT: time spent in lit side	Significant effects: CSDS: *F*_1,36_ = 8.73, *p* < 0.01 vehicle/MPL: *F*_1,36_ = 7.37, *p* < 0.05 CSDS × vehicle/MPL interaction: *F*_1,36_ = 15.48, *p* < 0.001
Figure 3F	LDT: number of total transitions in both lit and dark side	No significant effects: CSDS: *F*_1,36_ = 0.57, *p* = 0.46 vehicle/MPL: *F*_1,36_ = 0.16, *p* = 0.69 CSDS × vehicle/MPL interaction: *F*_1,36_ = 0.57, *p* = 0.46
Figure 3G	OFT: time spent in the center region of the open field	Significant effects: CSDS: *F*_1,36_ = 16.43, *p* < 0.001 vehicle/MPL: *F*_1,36_ = 8.48, *p* < 0.01 CSDS × vehicle/MPL interaction: *F*_1,36_ = 14.81, *p* < 0.001
Figure 3H	OFT: total distance	No significant effects: CSDS: *F*_1,36_ = 0.0009, *p* = 0.98 vehicle/MPL: *F*_1,36_ = 0.30, *p* = 0.59 CSDS × vehicle/MPL interaction: *F*_1,36_ = 0.03, *p* = 0.86
Figure 4		
Figure 4B	EPM test: time spent in open arms	Significant effects: CSDS: *F*_1,54_ = 11.65, *p* < 0.01 vehicle/MPL: *F*_2,54_ = 3.18, *p* < 0.05 CSDS × vehicle/MPL interaction: *F*_2,54_ = 3.57, *p* < 0.05
Figure 4C	EPM test: number of entries into open arms	Significant effects: CSDS: *F*_1,54_ = 18.51, *p* < 0.001 vehicle/MPL: *F*_2,54_ = 4.09, *p* < 0.05 CSDS × vehicle/MPL interaction: *F*_2,54_ = 6.87, *p* < 0.01
Figure 4D	EPM test: number of entries into total arms	No significant effects: CSDS: *F*_1,54_ = 0.12, *p* = 0.73 vehicle/MPL: *F*_2,54_ = 0.49, *p* = 0.61 CSDS × vehicle/MPL interaction: *F*_2,54_ = 0.11, *p* = 0.89
Figure 4E	LDT: time spent in lit side	Significant effects: CSDS: *F*_1,54_ = 19.37, *p* < 0.001 vehicle/MPL: *F*_2,54_ = 3.54, *p* < 0.05 CSDS × vehicle/MPL interaction: *F*_2,54_ = 6.07, *p* < 0.01
Figure 4F	LDT: number of total transitions in both lit and dark side	No significant effects: CSDS: *F*_1,54_ = 0.02, *p* = 0.90 vehicle/MPL: *F*_2,54_ = 0.26, *p* = 0.77 CSDS × vehicle/MPL interaction: *F*_2,54_ = 0.56, *p* = 0.58
Figure 4G	OFT: time spent in the center region of the open field	Significant effects: CSDS: *F*_1,54_ = 19.80, *p* < 0.001 vehicle/MPL: *F*_2,54_ = 6.21, *p* < 0.01 CSDS × vehicle/MPL interaction: *F*_2,54_ = 3.55, *p* < 0.05
Figure 4H	OFT: total distance	No significant effects: CSDS: *F*_1,54_ = 0.02, *p* = 0.89 vehicle/MPL: *F*_2,54_ = 0.49, *p* = 0.61 CSDS × vehicle/MPL interaction: *F*_2,54_ = 0.07, *p* = 0.94
Figure 5		
Figure 5A	Hippocampal IL-1β	Significant effects: CSDS: *F*_1,36_ = 27.89, *p* < 0.001 vehicle/MPL: *F*_1,36_ = 9.79, *p* < 0.01 CSDS × vehicle/MPL interaction: *F*_1,36_ = 11.58, *p* < 0.01
Figure 5B	Hippocampal IL-6	Significant effects: CSDS: *F*_1,36_ = 28.76, *p* < 0.001 vehicle/MPL: *F*_1,36_ = 8.14, *p* < 0.01 CSDS × vehicle/MPL interaction: *F*_1,36_ = 9.54, *p* < 0.01
Figure 5C	Hippocampal TNF-α	Significant effects: CSDS: *F*_1,36_ = 10.38, *p* < 0.01 vehicle/MPL: *F*_1,36_ = 4.48, *p* < 0.05 CSDS × vehicle/MPL interaction: *F*_1,36_ = 4.96, *p* < 0.05
Figure 5D	Cortical IL-1β	Significant effects: CSDS: *F*_1,36_ = 25.63, *p* < 0.001 vehicle/MPL: *F*_1,36_ = 9.55, *p* < 0.01 CSDS × vehicle/MPL interaction: *F*_1,36_ = 6.74, *p* < 0.05
Figure 5E	Cortical IL-6	Significant effects: CSDS: *F*_1,36_ = 12.93, *p* < 0.001 vehicle/MPL: *F*_1,36_ = 5.85, *p* < 0.05 CSDS × vehicle/MPL interaction: *F*_1,36_ = 7.08, *p* < 0.05
Figure 5F	Cortical TNF-α	Significant effects: CSDS: *F*_1,36_ = 14.57, *p* < 0.001 vehicle/MPL: *F*_1,36_ = 4.16, *p* < 0.05 CSDS × vehicle/MPL interaction: *F*_1,36_ = 6.93, *p* < 0.05
Figure 6		
Figure 6A	Hippocampal IL-6	Significant effects: vehicle/MPL: *F*_1,28_ = 50.33, *p* < 0.001 minocycline: *F*_1,28_ = 26.69, *p* < 0.001 vehicle/MPL × minocycline interaction: *F*_1,28_ = 21.18, *p* < 0.001
Figure 6A	Hippocampal IL-1β	Significant effects: vehicle/MPL: *F*_1,28_ = 39.74, *p* < 0.001 minocycline: *F*_1,28_ = 21.01, *p* < 0.001 vehicle/MPL × minocycline interaction: *F*_1,28_ = 18.68, *p* < 0.001
Figure 6B	Cortical IL-6	Significant effects: vehicle/MPL: *F*_1,28_ = 35.22, *p* < 0.001 minocycline: *F*_1,28_ = 18.53, *p* < 0.001 vehicle/MPL × minocycline interaction: *F*_1,28_ = 16.03, *p* < 0.001
Figure 6B	Cortical IL-1β	Significant effects: vehicle/MPL: *F*_1,28_ = 32.60, *p* < 0.001 minocycline: *F*_1,28_ = 21.23, *p* < 0.001 vehicle/MPL × minocycline interaction: *F*_1,28_ = 16.66, *p* < 0.001
Figure 6D	EPM test: time spent in open arms	Significant effects: vehicle/MPL: *F*_1,36_ = 11.15, *p* < 0.01 minocycline: *F*_1,36_ = 7.66, *p* < 0.01 vehicle/MPL × minocycline interaction: *F*_1,36_ = 5.20, *p* < 0.05
Figure 6E	EPM test: number of entries into open arms	Significant effects: vehicle/MPL: *F*_1,36_ = 15.24, *p* < 0.001 minocycline: *F*_1,36_ = 6.48, *p* < 0.05 vehicle/MPL × minocycline interaction: *F*_1,36_ = 5.65, *p* < 0.01
Figure 6F	EPM test: number of entries into total arms	No significant effects: vehicle/MPL: *F*_1,36_ = 1.35, *p* = 0.25 minocycline: *F*_1,36_ = 0.13, *p* = 0.72 vehicle/MPL × minocycline interaction: *F*_1,36_ = 0.39, *p* = 0.54
Figure 6G	LDT: time spent in lit side	Significant effects: vehicle/MPL: *F*_1,36_ = 7.55, *p* < 0.01 minocycline: *F*_1,36_ = 10.60, *p* < 0.01 vehicle/MPL × minocycline interaction: *F*_1,36_ = 5.74, *p* < 0.05
Figure 6H	LDT: number of total transitions in both lit and dark side	No significant effects: vehicle/MPL: *F*_1,36_ = 0.008, *p* = 0.93 minocycline: *F*_1,36_ = 0.60, *p* = 0.38 vehicle/MPL × minocycline interaction: *F*_1,36_ = 0.10, *p* = 0.76
Figure 6I	OFT: time spent in the center region of the open field	Significant effects: vehicle/MPL: *F*_1,36_ = 9.39, *p* < 0.01 minocycline: *F*_1,36_ = 6.78, *p* < 0.05 vehicle/MPL × minocycline interaction: *F*_1,36_ = 4.59, *p* < 0.05
Figure 6J	OFT: total distance	No significant effects: vehicle/MPL: *F*_1,36_ = 0.78, *p* = 0.38 minocycline: *F*_1,36_ = 0.42, *p* = 0.52 vehicle/MPL × minocycline interaction: *F*_1,36_ = 0.44, *p* = 0.51
Figure 7		
Figure 7A	Hippocampal IL-1β	Significant effects: vehicle/MPL: *F*_1,36_ = 10.07, *p* < 0.01 minocycline: *F*_1,36_ = 6.25, *p* < 0.05 vehicle/MPL × minocycline interaction: *F*_1,36_ = 10.65, *p* < 0.01
Figure 7B	Hippocampal IL-6	Significant effects: vehicle/MPL: *F*_1,36_ = 4.68, *p* < 0.05 minocycline: *F*_1,36_ = 5.64, *p* < 0.05 vehicle/MPL × minocycline interaction: *F*_1,36_ = 10.12, *p* < 0.01
Figure 7C	Hippocampal TNF-α	Significant effects: vehicle/MPL: *F*_1,36_ = 7.96, *p* < 0.01 minocycline: *F*_1,36_ = 6.07, *p* < 0.05 vehicle/MPL × minocycline interaction: *F*_1,36_ = 11.44, *p* < 0.01
Figure 7D	Cortical IL-1β	Significant effects: vehicle/MPL: *F*_1,36_ = 12.13, *p* < 0.01 minocycline: *F*_1,36_ = 5.70, *p* < 0.05 vehicle/MPL × minocycline interaction: *F*_1,36_ = 15.00, *p* < 0.001
Figure 7E	Cortical IL-6	Significant effects: vehicle/MPL: *F*_1,36_ = 13.01, *p* < 0.001 minocycline: *F*_1,36_ = 4.76, *p* < 0.05 vehicle/MPL × minocycline interaction: *F*_1,36_ = 7.06, *p* < 0.05
Figure 7F	Cortical TNF-α	Significant effects: vehicle/MPL: *F*_1,36_ = 11.16, *p* < 0.01 minocycline: *F*_1,36_ = 4.29, *p* < 0.05 vehicle/MPL × minocycline interaction: *F*_1,36_ = 9.41, *p* < 0.01

## Results

### MPL prevents the development of anxiety-like behaviors in CSDS mice

In our initial experiments, the dosages of 200, 400, and 800 μg/kg were selected to investigate the effect of MPL pre-injection on CSDS-induced anxiety-like behaviors (Fig. 1A). In the EPM test, we found that a single MPL injection 1 day before stress exposure at the dose of 400 and 800 μg/kg but not 200 μg/kg prevented CSDS-induced reductions in the time spent in open arms (Fig. 1B) and the number of entries into open arms (Fig. 1C) with no changes in the number of entries into total arms in mice treated without or with CSDS and/or MPL (Fig. 1D). In the LDT, the single MPL injection 1 day before stress exposure at the dose of 400 and 800 μg/kg was found to prevent CSDS-induced decreases in the time spent in lit side (Fig. 1E) with no changes in the number of total transitions in both lit and dark side in mice treated without or with CSDS and/or MPL (Fig. 1F). In the OFT, the single MPL injection 1 day before stress exposure at the dose of 400 and 800 μg/kg prevented CSDS-induced decrease in the time spent in the center region of the open field in the OFT (Fig. 1G) with no changes of total distance in mice treated without or with CSDS and/or MPL (Fig. 1H). Further analysis showed that injection of MPL at all selected doses did not affect the behavioral phenotypes mentioned above in stress-naïve mice in the EPM test (Fig. 1B‒D), LDT (Fig. E, F), and OFT (Fig. 1G, H). Considering that the preventive effect of MPL on chronic stress-induced anxiety-like behaviors peaked at the dose of 400 μg/kg, the 400 μg/kg dosage was used in following experiments.

### Influence of time-interval on the preventive effect of MPL on anxiety-like behaviors in CSDS mice

Next, we evaluated the effect of different interval time on the preventive effect of MPL on CSDS-induced anxiety-like behaviors (Fig. 2A). In the EPM test in model 1, a single MPL injection (400 μg/kg) 1 day before stress exposure was found to prevent CSDS-induced reductions in the time spent in open arms (Fig. 2B) and the number of entries into open arms (Fig. 2C) with no changes in the number of entries into total arms in mice treated without or with CSDS and/or MPL (Fig. 2D). In the LDT in model 1, the single MPL injection (400 μg/kg) 1 day before stress exposure prevented CSDS-induced decrease in the time spent in lit side (Fig. 2E) with no changes in the number of total transitions in both lit and dark side in mice treated without or with CSDS and/or MPL (Fig. 2F). In the OFT in model 1, the single MPL injection 1 day before stress exposure at a dose of 400 μg/kg was found to prevent CSDS-induced decrease in the time spent in the center region of the open field (Fig. 2G) with no changes of total distance in mice treated without or with CSDS and/or MPL (Fig. 2H).

In the EPM test in model 2, we found that a single MPL injection (400 μg/kg) 5 days before stress exposure prevented CSDS-induced reductions in the time spent in open arms (Fig. 2B) and the number of entries into open arms (Fig. 2C) with no changes in the number of entries into total arms in mice treated without or with CSDS and/or MPL (Fig. 2D). In the LDT in model 2, the single MPL injection (400 μg/kg) 5 days before stress exposure prevented CSDS-induced decrease in the time spent in lit side (Fig. 2E) with no changes in the number of total transitions in both lit and dark side in mice treated without or with CSDS and/or MPL (Fig. 2F). In the OFT in model 2, the single MPL injection 5 days before stress exposure at a dose of 400 μg/kg prevented CSDS-induced decrease in the time spent in the center region of the open field (Fig. 2G) with no changes of total distance in mice treated without or with CSDS and/or MPL (Fig. 2H).

In model 3, the chronically stressed mice who received a single MPL injection (400 μg/kg) 10 days before stress exposure still displayed anxiety-like behaviors in the EPM test (Fig. 2B, C), LDT (Fig. 2E), and OFT (Fig. 2G), with no changes in the number of entries into total arms in the EPM test (Fig. 2D), the number of total transitions in both lit and dark side in the LDT (Fig. 2F), and the total distance of mice in the OFT (Fig. 2H), suggesting that if the interval time between MPL pretreatment and stress exposure was extended to 10 days (model 3), the MPL injection no longer elicited a prophylactic effect on CSDS-induced anxiety-like behaviors.

### Re-observed preventive effect of a second MPL injection 10 days after the first MPL injection on CSDS-induced anxiety-like behaviors

In following experiments, we evaluated whether a second MPL injection 10 days after the first MPL injection can produce a preventive effect (Fig. 3A). In the EPM test, we found that a second MPL injection 10 days after the first MPL injection prevented CSDS-induced reductions in the time spent in open arms (Fig. 3B) and the number of entries in open arms (Fig. 3C) with no changes in the number of entries into total arms in mice treated without or with CSDS and/or MPL (Fig. 3D). In the LDT, the second MPL injection 10 days after the first MPL injection was found to prevent CSDS-induced decrease in the time spent in lit side (Fig. 3E) with no changes in the number of total transitions in both lit and dark side in mice treated without or with CSDS and/or MPL (Fig. 3F). In the OFT, the second MPL injection 10 days after the first MPL injection prevented CSDS-induced decrease in the time spent in the center region of the open field (Fig. 3G) with no changes of total distance in mice treated without or with CSDS and/or MPL (Fig. 3H).

### Effect of repeated MPL injection 10 days before stress exposure on CSDS-induced anxiety-like behaviors

We also investigated whether repeated MPL injections (4 × injections, Fig. 4A) 10 days before stress exposure can produce preventive effect on CSDS-induced anxiety-like behaviors. In the EPM test, we found that the 4 × MPL injections (4 consecutive days, 400 μg/kg) prevented CSDS-induced reductions in the time spent in open arms (Fig. 4B) and the number of entries in the open arms (Fig. 4C) with no changes in the number of entries into total arms in mice treated without or with CSDS and/or MPL (Fig. 4D). In the LDT, the 4 × MPL injections prevented CSDS-induced decrease in the time spent in lit side (Fig. 4E) with no changes in the number of total transitions in both lit and dark side in mice treated without or with CSDS and/or MPL (Fig. 4F). In the OFT, the 4 × MPL injections prevented CSDS-induced decrease in the time spent in the center region of the open field (Fig. 4G) with no changes of total distance in mice treated without or with CSDS and/or MPL (Fig. 4H).

### Effect of MPL pre-injection on CSDS-induced neuroinflammatory response in the brain

Considering that innate immune pre-stimulation can induce neuroprotective effect by reducing neuroinflammation [16, 20, 21], we then evaluated whether a single MPL injection 1 day before stress exposure can prevent the overproduction of pro-inflammatory cytokines in the brain in chronically stressed mice. Results showed that the single MPL injection (400 μg/kg) 1 day before stress exposure prevented CSDS-induced increases in the levels of IL-1β (Fig. 5A), IL-6 (Fig. 5B), and TNF-α (Fig. 5C) in the hippocampus. Similarly, the single MPL injection (400 μg/kg) 1 day before stress exposure was also found to prevent the abnormal increases of IL-1β (Fig. 5D), IL-6 (Fig. 5E), and TNF-α (Fig. 5F) levels in the medial prefrontal cortex in CSDS mice.

### Minocycline pretreatment abrogates MPL-induced prevention of CSDS-induced anxiety-like behaviors

One of the major consequences of MPL pre-injection is the induction of innate immune activation. Here, we focused on a question that whether microglial activation mediates the preventive effect of MPL on CSDS-induced anxiety-like behaviors. To answer this question, we first investigated the effect of minocycline pretreatment on acute MPL injection-induced neuroinflammatory responses in the brain. Results showed that 40 mg/kg of minocycline pretreatment prevented acute MPL injection (5 h, 400 μg/kg)-induced increases in the expression levels of IL-6 and IL-1β mRNA in the hippocampus (Fig. 6A) and medial prefrontal cortex (Fig. 6B). This demonstrated that the central immune activation triggered by acute MPL injection could be blocked by minocycline pretreatment.

Then, we examined the changes in behavioral phenotypes in mice treated without or with minocycline, MPL, and/or CSDS (Fig. 6C). In the EPM test, we found that minocycline pretreatment (40 mg/kg) abrogated the preventive effect of a single MPL injection (400 μg/kg) 1 day before stress exposure on CSDS-induced reductions in the time spent in open arms (Fig. 6D) and the number of entries in open arms (Fig. 6E) in mice with no changes in the number of entries into total arms (Fig. 6F). In the LDT, minocycline pretreatment (40 mg/kg) was found to abrogate the preventive effect of the single MPL injection (400 μg/kg) 1 day before stress exposure on CSDS-induced reductions in the time spent in lit side (Fig. 6G) in mice with no changes in the number of total transitions in both lit and dark side (Fig. 6H). In the OFT, minocycline pretreatment (40 mg/kg/day) abrogated the preventive effect of the single MPL injection (400 μg/kg) 1 day before stress exposure on CSDS-induced reductions in the time spent in the center region of the open field (Fig. 6I) in mice with no changes of total distance (Fig. 6J).

### Minocycline pretreatment abrogates MPL-induced prevention of CSDS-induced neuroinflammatory response in the brain

Finally, we evaluated whether minocycline pretreatment could abrogate the preventive effect of MPL pretreatment on CSDS-induced neuroinflammatory responses in the brain. As shown in Fig. 7A–C, minocycline pretreatment at the dose of 40 mg/kg abrogated the preventive effect of the single MPL injection (400 μg/kg) 1 day before stress exposure on CSDS-induced increases in the levels of IL-1β (Fig. 7A), IL-6 (Fig. 7B), and TNF-α (Fig. 7C) in the hippocampus. Similarly, minocycline pretreatment at the dose of 40 mg/kg also abrogated the preventive effect of the single MPL injection (400 μg/kg) 1 day before stress exposure on CSDS-induced increases in the levels of IL-1β (Fig. 7D), IL-6 (Fig. 7E), and TNF-α (Fig. 7F) in the medial prefrontal cortex.

## Discussion

One of the major findings in the present study is that a single MPL injection 1 day before stress exposure prevented 10 days of social defeat stress-induced anxiety-like behaviors in mice, and no anxiety-inducing effect of MPL was observed at any dosage in stress-naïve mice. This indicated that the application of MPL, a molecule which lacks undesirable effects of LPS [25, 26], may be relatively safe for the prevention of chronic stress-induced anxiety-like behaviors in animals, and compounds which mobilize the innate immune response could be developed as novel drugs for the prevention of anxiety-associated behaviors in humans.

In daily life, the anxiety-associated behaviors can be induced by many factors, such as repeated social defeat stress [6, 30], high-fat intake [31], and metabolic disturbance [32]. Rocha-Gomes et al. reported that a low dose of LPS injection during mouse gestation can induce a preventive effect on anxiety-like behaviors and neuroinflammatory responses in the amygdala in high-fat-fed dam’s adolescent offspring [33]. This demonstrated a possible preventive effect of innate immune pre-stimulation on the development of anxiety-like behaviors in animals. Our study’s findings showed a specific effect of MPL pre-injection on CSDS-induced anxiety-like behaviors in adult mice. Whether MPL can produce similar preventive effect on anxiety-like behaviors induced by other factors remains to be determined by future studies.

In past studies, innate immune stimulants including LPS and colony stimulating factor are usually used to study the preventive effect of innate immune pre-stimulation on neuronal damage and neuroinflammatory responses in central nervous system disorders. These agents, however, can also induce detrimental effects on body functions [22, 23, 34]. In the present study, we selected MPL, a compound that possesses fewer undesirable actions and a significant therapeutic window compared to LPS [25, 26], to study the preventive effect of innate immune pre-stimulation on chronic stress-induced anxiety-like behaviors. MPL is commercialized as a vaccine adjuvant [35, 36] and can induce neuroprotective effects. Pre-administration of animals with a low dose of MPL has been demonstrated to protect the neurons against ischemic stimuli [37] and reduce seizure severity induced by traumatic brain injury or pilocarpine by reducing the over-production of pro-inflammatory cytokines in the brain [38, 39]. Our findings extend the pharmacological effect of MPL as a vaccine-like drug for the prevention of CSDS-induced anxiety-like behaviors.

The results in Fig. 1 showed that as the increase in administration dosage, the preventive effect of MPL enhanced progressively, suggesting that we should select a proper dosage of MPL to induce an optimized preventive effect on chronic stress-induced anxiety-like behaviors. We also found that the preventive effect of the single dose of MPL pre-injection on CSDS-induced anxiety-like behaviors vanished with the extension of the observation time after administering MPL: the single MPL injection 1 day and 5 days before stress exposure prevented the development of anxiety-like behaviors in CSDS mice, but if the interval time between MPL injection and stress exposure was prolonged to 10 days, the MPL pre-injection failed to produce similar effect. This could be due to a possibility that the long-term interval between MPL pre-injection and stress exposure induces a loss of factors that can mediate the neuroprotective effect of MPL. This hypothesis should be examined by future studies.

If the preventive effect of MPL on anxiety vanishes rapidly, the application of MPL in anxiety prevention would be restricted. To solve this issue, we addressed whether a second MPL injection after the disappearance of the neuroprotective effect of the first MPL injection can still induce preventive effect on chronic stress-induced anxiety-like behaviors. We found that a second MPL injection 10 days after the first MPL injection rendered the mice against CSDS-induced anxiety-like behaviors. We thus assumed that similar with traditional vaccines that are used to prevent pathogen infections, the disappeared preventive effect of MPL pre-injection on chronic stress-induced anxiety-like behaviors could be re-acquired by subsequent repeated injections. As the preventive effect of MPL on CSDS-induced anxiety-like behaviors increased with the increasing of MPL dosage, we questioned whether a consecutive and repeated MPL injection 10 days before stress exposure can produce similar tolerance effect. As expected, 4 × MPL injections 10 days before stress exposure were found to prevent CSDS-induced anxiety-like behaviors, demonstrating that consolidated immunization by increasing the administration times of MPL can prolong the interval time during which tolerance is maintained against the later stress exposure. Then, why it is the multiple injections but not a single MPL injection 10 days before stress exposure that induces preventive effect on anxiety-like behaviors? An explanation could be that repeated MPL injections induce a more potent activation of the innate immune cells than the single MPL injection. In future studies, we should clarify which molecules mediate the long-term effect of repeated MPL injections on chronic stress-induced anxiety-like behaviors.

Although MPL lacks undesirable effects of LPS [25], it can still induce immune cell activation [40, 41]. In the current study, minocycline pretreatment was found to suppress acute MPL injection-induced neuroinflammatory responses in the brain and simultaneously abrogate the preventive effect of MPL pre-injection on CSDS-induced anxiety-like behaviors in mice. This demonstrated that the immune cell activation was necessary to facilitate the preventive effect of MPL on CSDS-induced anxiety-like behaviors. Currently, we still do not know which types of immune cells mediate the initial activation of the innate immune response by MPL injection. The microglia may produce a great contribution in that process, as (i) studies involving the bone-marrow chimeric mice have reported that the presence of TLR4 or myeloid differentiation factor 88 (MyD88), a critical adaptor protein for most TLR, in microglia but not in hematogenous immune cells, is required for the transduction of the neuroprotective effect of LPS pretreatment [42, 43]; (ii) direct depletion of microglia can abrogate the preventive effect of a low dose of LPS preconditioning on pilocarpine-induced seizure [44] or chronic stress-induced depression-like behaviors in mice [20]; and (iii) specifically activation of the TLR9 signaling in microglia has been shown to reduce seizure-induced cognitive decline and recurrent seizure severity [45]. However, the role of the other immune cells in the preventive effect of MPL on CSDS-induced anxiety-like behaviors cannot be precluded completely, as the depletion of the in vivo regulatory T cells has been reported to abrogate the preventive effect of a heat-killed preparation of Mycobacterium vaccae, an immuno-regulatory environmental microorganism, on stress-induced anxiety- and fear-like behaviors in mice [46], and more importantly the crosstalk between microglia and neurons and/or astrocytes appears to be necessary for the prevention of pro-inflammatory cytokine production by LPS preconditioning [47]. In future studies, we should clarify the cellular basis for the preventive effect of MPL on chronic stress-induced anxiety-like behaviors.

Our studies also showed that the preventive effect of MPL pre-injection on CSDS-induced anxiety-like behaviors was associated with the reduction of the production of pro-inflammatory cytokines in the hippocampus and medial prefrontal cortex. This finding demonstrated that the innate immune stimulation induced by MPL injection can prevent the progression of neuroinflammatory response in chronically stressed animals. The accumulation of pro-inflammatory cytokines in the brain could be due to the over-activation of the in situ microglia. Previously published in vitro studies have reported that LPS pretreatment can prevent the production of pro-inflammatory cytokines in primary cultured microglia by inducing an epigenetically regulated immuno-suppressive phenotype [48]. Furthermore, the induction of interferon regulatory factor 3 is considered to be necessary for the neuroprotective actions of LPS preconditioning [49]. Whether these mechanisms can mediate the preventive effect of MPL pretreatment on CSDS-induced anxiety-like behaviors should be examined in future studies. As peripheral macrophages which are recruited to the brain upon stress stimulation can also drive behavioral deficits by producing high levels of pro-inflammatory cytokines [50, 51], we should also clarify the roles of these cells in the preventive effect of MPL on CSDS-induced anxiety-like behaviors.

## Conclusion

Our results showed that MPL pre-injection can prevent CSDS-induced anxiety-like behaviors and neuroinflammatory responses in mice. An interval of 10 days between MPL injection and stress exposure abolished the preventive effect of MPL on CSDS-induced anxiety-like behaviors, which was rescued with consolidated immunization by a second MPL injection 10 days after the first MPL injection or 4 × MPL injections 1 day before stress exposure. As MPL is used as a vaccine adjuvant in clinic [35, 36] and shows little toxicities compared to its parent molecule LPS [52], our findings may provide a promising alternative for the development of MPL as novel drugs for the prevention of anxiety-associated behaviors.

## Data Availability

The data sets used and/or analyzed during the current study are available from the corresponding author on reasonable request.

## Abbreviations

ANOVA: Analysis of variance
CSDS: Chronic social defeat stress
DMSO: Dimethyl sulfoxide
EPM: Elevated plus maze
GAPDH: Glyceraldehyde-3-phosphate dehydrogenase
IL-1β: Interleukin-1β
IRF: Interferon regulatory factor
JMJD3: Jumonji domain-containing protein D3
LDT: Light–dark test
LPS: Lipopolysaccharide
MPL: Monophosphoryl lipid A
MyD88: Myeloid differentiation factor 88
OFT: Open field test
TLR2/4: Toll-like receptor 2/4
